# Management of infodemics in outbreaks or health crises: a systematic review

**DOI:** 10.3389/fpubh.2024.1343902

**Published:** 2024-03-15

**Authors:** Lamis Abuhaloob, Tina D. Purnat, Celine Tabche, Zeenah Atwan, Elizabeth Dubois, Salman Rawaf

**Affiliations:** ^1^Faculty of Medicine, WHO Collaborating Centre for Public Health Education and Training, School of Public Health, Imperial College London, London, United Kingdom; ^2^University of Memphis School of Public Health, Memphis, TN, United States; ^3^Department of Microbiology, Faculty of Medicine, University of Al-Basra, Al-Basra, Iraq

**Keywords:** infodemic, infodemic management, infodemiology, COVID-19, outbreak, health crisis, health emergency, misinformation

## Abstract

**Introduction:**

The World Health Organization (WHO) defined an infodemic as an overabundance of information, accurate or not, in the digital and physical space, accompanying an acute health event such as an outbreak or epidemic. It can impact people’s risk perceptions, trust, and confidence in the health system, and health workers. As an immediate response, the WHO developed the infodemic management (IM) frameworks, research agenda, intervention frameworks, competencies, and processes for reference by health authorities.

**Objective:**

This systematic review explored the response to and during acute health events by health authorities and other organizations operating in health. It also assessed the effectiveness of the current interventions.

**Methods:**

On 26 June 2023, an online database search included Medline (Ovid), Embase, Cochrane Library, Scopus, Epistemonikos, and the WHO website. It included English-only, peer-reviewed studies or reports covering IM processes applied by health organizations that reported their effectiveness. There was no restriction on publication dates. Two independent reviewers conducted all screening, inclusion, and quality assessments, and a third reviewer arbitrated any disagreement between the two reviewers.

**Results:**

Reviewers identified 945 records. After a final assessment, 29 studies were included in the review and were published between 2021 and 2023. Some countries (Pakistan, Yemen, Spain, Italy, Hong Kong, Japan, South Korea, Singapore, United Kingdom, United States, New Zealand, Finland, South Korea, and Russia) applied different methods of IM to people’s behaviors. These included but were not limited to launching media and TV conservations, using web and scientific database searches, posting science-based COVID-19 information, implementing online surveys, and creating an innovative ecosystem of digital tools, and an Early AI-supported response with Social Listening (EARS) platform. Most of the interventions were effective in containing the harmful effects of COVID-19 infodemic. However, the quality of the evidence was not robust.

**Discussion:**

Most of the infodemic interventions applied during COVID-19 fall within the recommended actions of the WHO IM ecosystem. As a result, the study suggests that more research is needed into the challenges facing health systems in different operational environments and country contexts in relation to designing, implementing, and evaluating IM interventions, strategies, policies, and systems.

## 1 Introduction

### 1.1 Infodemics and the health system

The WHO defined an infodemic as an overabundance of information, accurate or not, in the digital and physical space, accompanying an acute health event such as an outbreak or epidemic (1–4). An infodemic consists of accurate, inaccurate, and outdated health information, information voids, as well as narratives and mis- and disinformation.

When acute health events occur, the information environment changes—people actively search for and share health information. The government is actively communicating on a particular topic and other experts contribute to the discussion of the subject in society. Communities who are not usually interested in health are now talking about it, and media and fact-checkers cover the topic of health more. In the uncertainty of an emergency, and often with evolving scientific knowledge about the topic, the chaotic information environment can make it difficult for people to find health information they search for, and need to protect themselves and their families, irrespective of their health literacy (1). In addition, a chaotic information environment, coupled with limits in access to health services and health diagnostics, therapeutics and vaccines. Those together with individual socioeconomic drivers and aspects of health and digital information literacies can limit the adherence to recommended health guidance and public health and social measures, and uptake of diagnostics and vaccine service (4–8).

Infodemics impact all levels of society: individual, family, community, health system, government, and society, and can lead to a variety of harms. Such harms include skewed risk perception and delayed healthcare seeking, victimization and stigmatization of vulnerable populations, panic buying, and falling for deceptive marketing. Mistrust in the government, health system, health workers, public health, social and medical countermeasures, lead to low adherence to recommended health guidance, anxiety, and stress (1, 9, 10).

### 1.2 Infodemic management and WHO infodemic management program

Infodemic management is the systematic use of risk- and evidence-based analysis and approaches to promote a healthier information environment and resilience against infodemics negative impacts on health behaviors during health emergencies. Systematic application of infodemic management approaches can mitigate the harm from infodemics during emergencies and promote resilience to infodemics and health misinformation, especially in populations experiencing inequities and vulnerabilities (4). During the COVID-19 pandemic, the WHO set up a toolbox of infodemic management interventions, promoting the science of infodemiology, professionalization of infodemic management practice, and partnerships across all of society (such as with civil society, media, private sector, and multilateral and international organizations) (11). This was described through a whole-of-society framework for responding to the COVID-19 infodemic and 50 actions that can be taken across society to do so (2), along with four pillars: (1) Identify evidence, (2) Translate knowledge and science, (3) amplify action, (4) quantify impact.

Based on that, to tackle infodemics during the COVID-19 pandemic, the WHO infodemic management team conducted global online consultations and conferences on various aspects of prioritizing infodemiological research, sharing experiences and tools, developing capacities and competency framework for infodemic management, to advance metrics and frameworks (12–15). Operationally, WHO developed partnerships with search, social, and digital companies like Facebook, Google, Tencent, Baidu, Twitter, TikTok, Weibo, Pinterest, and YouTube to promote distribution of WHO’s health content. Regionally, Africa Infodemic Response Alliance, a partnership hosted by WHO Regional Office for Africa, was established to facilitate social listening and rapid response to misinformation and infodemic impacts on communities (16, 17). As part of the WHO incident management response, the WHO infodemic management developed and implemented novel analytical approaches in over 18 languages for weekly social listening, integrated analysis, and infodemic insights generation. In addition to finding information voids, circulating narratives on mis- and disinformation, they used these to understand peoples’ questions, concerns, and provide recommendations for actions to address them (1, 14, 16).

Through various activities, the WHO identified interdisciplinary approaches and frameworks to measure the burden of infodemics (2, 4, 7, 9, 18). Four categories of intervention that the WHO recommends managing infodemics. These are (1) listening questions, concerns, information voids, and circulating narratives including mis- and disinformation, (2) communicating science and risk, (3) promoting resilience to infodemics and health misinformation, and (4) engaging and empowering communities (9). The WHO recommends that successful infodemic management should be embedded within health authority’s routine functions and structures (4).

As the health systems globally have moved to restore routine health services and recovery from the pandemic impacts, an effort has been made to integrate the lessons learned. These efforts involved new partnerships, and tools that were established during the pandemic into other emergency responses, the health system and into preparedness planning. For example, social listening infodemic insights and infodemic management have been included in the WHO toolkits for country preparedness and resilience planning. These include emerging threats for pandemic influenza preparedness and for response to influenza outbreaks in animals, WHO’s global architecture for health emergency prevention, preparedness, response and resilience, WHO and partners’ framework for vaccine demand promotion and integration for COVID-19 vaccination into routine immunization and primary health care, among others (19–24). While countries have reported to WHO conferences and trainings their infodemic management activities, health authorities have not yet extensively reported and published their experience in scientific literature, with Germany being the first (25, 26).

### 1.3 The gap in evidence related to infodemic management interventions

During the COVID-19 pandemic, many different strategies were designed and applied globally and in different settings to mitigate the harms from the COVID-19 infodemic and infodemics accompanying other outbreaks such as mpox, cholera, Ebola, measles, and diphtheria. The WHO recognized that there is a need to develop a comprehensive taxonomy of infodemic management interventions and outcomes and has convened an expert group to perform an evidence and gap map (27).

While this is ongoing, there is a lack of information on the practices in infodemic management in countries and different sectors of society. Thus, this systematic review aims to explore how health authorities and other organizations working in health have responded to the COVID-19 infodemic and assess the management effectiveness.

## 2 Methods

The main research question is, “Are infodemic management interventions that have been used during health crises effective?” Other questions to address are: Which infodemic management interventions, strategies and approaches have been used by health authorities to manage infodemics? Are current infodemic management strategies effective enough to mitigate harm from an infodemic?

To address these questions, a systematic search was conducted for primary and secondary literature in the databases (Embase^®^, WHO IRIS, Cochrane Library of Systematic Reviews, Scopus and Epistemonikos) and explored the reference lists of the included studies. We conducted the search on 26 June 2023. The search included MeSH terms and free text within each database, as illustrated in Box 1.

BOX 1 **Search string used within the database.**

**Table 1:** 

1 “management/ or manag*.mp,” 2 “misinformation/ or Misinformation.mp,” 3 “Misleading information.mp,” 4 “False information.mp,” 5 “gossip*.mp,” 6 “rumour*.mp,” 7 “hoax*.mp,” 8 “urban legend*.mp,” 9 “myth*.mp,” 10 “fallac*.mp,” 11 “infodemic/ or infodemiology/,” 12 “infodemic*.mp,” 13 “infodemiology.mp,” 14 “2 or 3 or 4 or 5 or 6 or 7 or 8 or 9 or 10 or 11 or 12 or 13,” 15 “Disease outbreak.mp. or epidemic/,” 16 “pandemic/ or pandemic*.mp,” 17 “epidemic*.mp,” 18 “15 or 16 or 17,” 19 “infodemic/ or Infodemic management.mp,” 20 “infodemiology/ or Infodemiology management.mp,” 21 “19 or 20,” 22 “1 and 14 and 18,” 23 “program effectiveness/ or effectiveness.mp,” 24 “health impact assessment/ or impact.mp. or program impact/,” 25 “23 or 24,” 26 “22 and 25,” 27 “21 or 26,” 28 “infodemiology/ or infodemi*.mp. or infodemic/,” 29 “manage*.mp,” 30 “28 and 29,” 31 “cris*.mp,” 32 “pandemic/ or pandemic.mp,” 33 “outbreak*.mp. or epidemic/,” 34 “31 or 32 or 33,” 35 “30 and 34,” 36 “27 or 35”

No time restriction was applied, and only studies published in English were included. After removing duplicates, two authors independently screened the title, abstract, and full text of articles and included eligible articles for evaluation. An independent third author resolved any disagreements. We performed the screening process in Covidence.

### 2.1 Selection of the literature

The following inclusion criteria were applied in the selection process: (1) Populations: any population that is experiencing an infodemic during outbreaks or health crises, (2) Interventions: peer-reviewed articles for any quasi-experiment, randomized control trial (RCT), interventions or programs aiming to manage infodemics (questions, concerns, information voids, narratives or mis- and disinformation) when preventing, preparing, or responding to acute health events, (3) Comparison: studies compared, evaluated, assessed, or planned spread, effect, or mitigating measures for infodemic during an outbreak, (4) Outcome: change in the harm from infodemic impact on the population of focus (e.g., change in health behaviors), (5) Study designs included observational and experimental studies, including RCT, cluster-RCT, and controlled before-after (CBA) studies.

The exclusion criteria included (1) Wrong study population: populations not targeted by infodemics during outbreaks or health crises, (2) Unreported study design: did not provide information about infodemic management interventions and/or their outcomes, (3) Unclear study outcome: did not record any information on the impact of infodemics management on the population, (4) Studies not published in English, (5) Study full text not found, (6) Duplicated paper.

### 2.2 Quality assessment

The study quality was assessed by two independent reviewers. CASP tools were used for assessing the qualities of experimental and observational studies and systematic reviews except for cross-sectional studies. The later study design was evaluated using JBI Critical Appraisal Tools.

### 2.3 Data extraction

The articles and reports that met the inclusion criteria were retained for data extraction and further analysis. Supplementary Table S1 shows the template developed to extract review-related information. The research team discussed and agreed upon the final characteristics of the table to extract data in this review. Two reviewers developed data extraction; one reviewer extracted the data to the template, and the second reviewer double-checked the extractions by the first reviewer. A third reviewer arbitrated any disagreement between the two reviewers.

### 2.4 Data analyses and synthesis

The synthesis included the categorization of relevant study findings. No attempt was made to perform a meta-analysis because of the high heterogeneity regarding population and intervention in the included studies. Finally, a descriptive-analytical method was used to present the review’s outcome. Conclusions and recommendations emerged from the findings and gaps identified by this review.

## 3 Results

**Figure 1:**
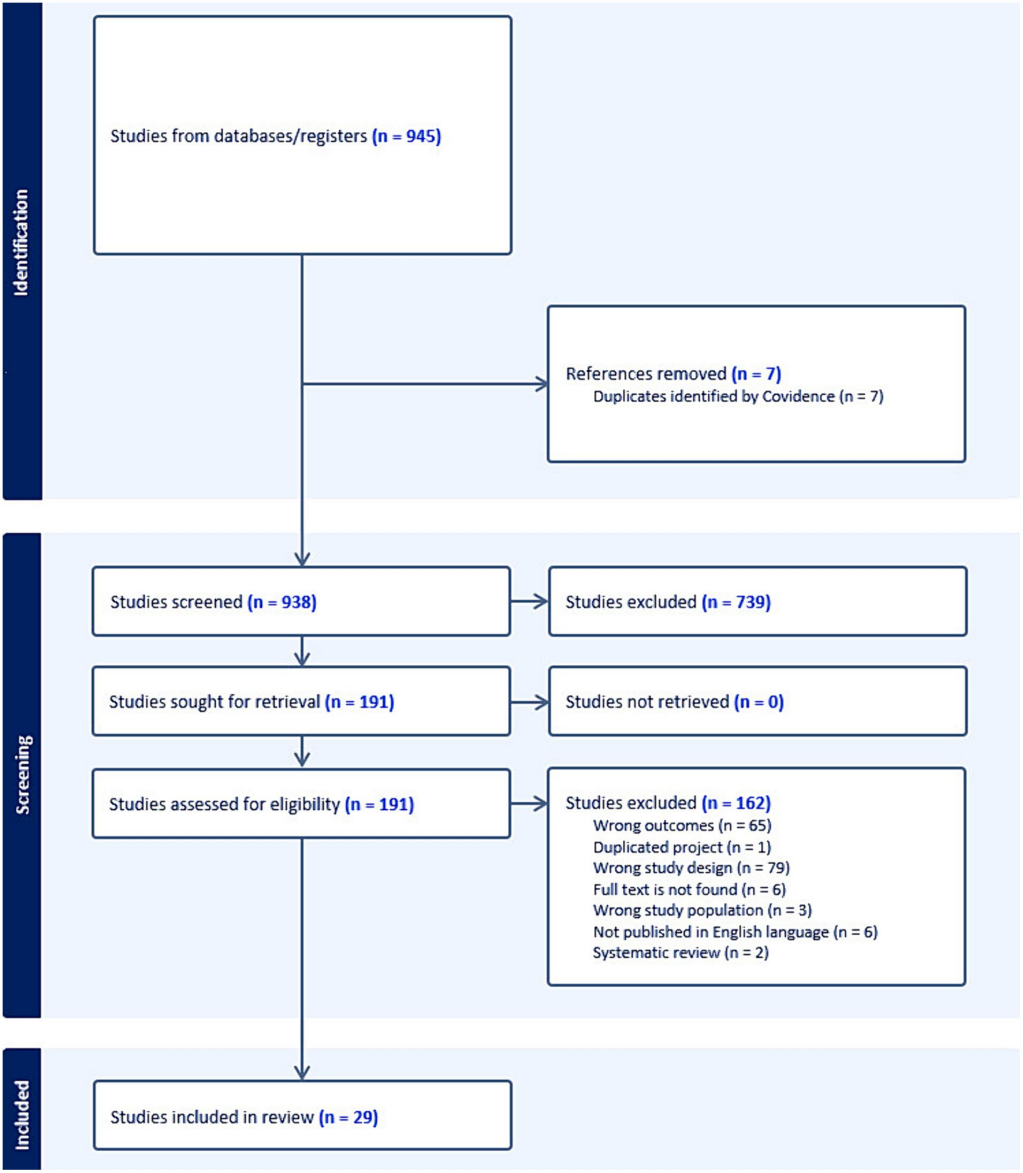
PRISMA for the literature selection process.

The database search identified 945 records. After removing duplications and screening abstracts according to our inclusion and exclusion criteria, our results came to 199 full-text studies. Finally, only 29 studies were included (Figure 1). Retained studies were published during the Pandemic between 2021 and 2023.

The studies were conducted in different countries, including Pakistan, Yemen, the Kingdom of Saudi Arabia, Spain, Italy, Hong Kong, Japan, South Korea, Singapore, the UK, the USA, New Zealand, Finland, South Korea, and Russia. The study designs of the included papers were observational and experimental studies. All studies tackled an aspect of the infodemic during the COVID-19 pandemic.

### 3.1 Sources of the infodemic

These studies focused on the analysis of different digital and physical environments and sources of health information such as social media posts and conservations, web, news, radio, TV talk shows, press conferences, national press, pre-print and peer-reviewed papers.

### 3.2 Infodemic impact on the health

A large adverse physical, social, political, and psychological harm from infodemics was detected by included studies on individual level (health behavior misaligned with recommended health guidance, fear or panic, and vaccine hesitancy), organizational level (misallocation of health resources and ineffective communication of risk), national level (unintended consequences of pandemic countermeasures and reduced cyber and information security, harm to public health) and global level (increased harm to mental health globally). On the other hand, it found that lower degree of government transparency accompanied with specific misinformation narratives lowered risk perception of COVID-19 and enacting recommended health behaviors.

### 3.3 Tools for managing infodemics

Methods used to manage the negative impact of infodemics were directed to the digital and physical platforms used for republishing and amplifying messages. Most of these interventions showed effectiveness in reducing harm from the infodemic. However, the overall quality of the evidence on effectiveness was only moderate. For example, Moretti et al. (28) reported an increase in the level of digital health literacy from 2.9 to 4.2 (*p* = 0.001) among Italian medical students after attending an infodemic course. This course trained students on the use of the “dottoremaeveroche” (DMEVC) web resource to assess the quality of medical information. However, the overall quality of evidence on effectiveness was only moderate. Identifying search keywords to learn about the outbreak or crises, is the initial tool for predicting the adverse effects on the individual, family, community and population health, as well as impacts at health systems and societal levels. The implemented interventions for infodemic management are summarized in Supplementary Table S2.

## 4 Discussion

This systematic review was conducted to explore how health authorities and other organizations working in health attempted to address the COVID-19 infodemic and assessed the effectiveness of these interventions. Although an infodemic consists of questions, concerns, information voids, and circulating narratives, including mis- and disinformation, most of the studies focused only on the misinformation element. It is estimated that only 0.2–28.8% of Twitter, Facebook, YouTube, and Instagram posts were of health-related misinformation (29, 30). This means that interventions that are reported in this review are dressing only a small part of the health information that is circulating at any one time in the information environment, and it is not comprehensive in its approach. Moreover, agreement is reported on the critical role of social media in addressing misinformation during crises (29), but again focuses only on digital environments and only on misinformation.

Although harmful impacts of health misinformation were experienced by a diverse set of health programs in the past, it was a niche area of academic research and practice in globally funded programs like immunization. Moreover, the terminology to describe the science and understanding of the complex challenge of the information environment on people’s risk perceptions and behaviors during acute health events changed and evolved over time. A common language, definitions of risk assessment approach, multilevel interventions and systems for health authorities can address it in a systematic, evidence-based way, only gained traction after 2020. This was associated with the evolution and investment into promoting the uptake of public health and social measures, and demand for treatments, diagnostics, and vaccines during the biggest pandemic the world experience in recent memory. The studies that are included in this systematic review were, therefore, unsurprisingly published between 2021 and 2023.

The WHO defined infodemic management as the systematic use of evidence-based risk analysis, and approaches to manage the infodemics and reduce any negative impact on health behaviors during emergencies (1, 9). Purnat et al. (9) discuss the infodemic management framework as the main component for health organizations to ensure that health system’s communications, services, actions, and interventions are meeting the needs of different populations and therefore enjoy the trust necessary to be resilient to information overload, unsettled science, inaccurate information and misinformation. One review, discussing social media platforms, suggests that together with improving people’s digital and health literacy, multi-sectorial action, governance policies, and implementing awareness campaigns, are all urgently needed (29).

Different countries responded to the COVID-19 pandemic and the associated infodemic by implementing digital interventions. For example, the Kingdom of Saudi Arabia hosted the Riyadh Global Digital Health Summit, which articulated nine recommendations for data communication and digital health that need to be adopted by the global health community to address future pandemics and health threats (31). The Riyadh Summit committee was looking to build on the declaration and to provide a resource and toolkit to develop digital health infrastructure at national and supranational levels to prepare for future health threats (31). The estimated budget for implementing such an initiative was equivalent to US$2.5 billion annually in Low- and Middle-Income Countries. In this review, only Yemen and Pakistan, as Low- and Middle-Income Countries, responded to the COVID-19 pandemic and infodemic (32, 33).

Around third of included studies were characterizing the flow of information by using Web and scientific database searches. Examples include developing a global search index based on Google Trends data and combining it with keywords to predict people’s offline attitudes and behaviors in the context of public health and social measures. It found that the most searched keywords to learn about the COVID-19 pandemic, during the first 6 months after the SARS-CoV-2 outbreak (1 January to 30 June 2020), were “pastCoVepidemics” and “presCoVpandemic” (34). In addition, it identified the predictors of people’s behavior toward public health measures, and they were “social distancing,” “wash hands,” “isolation,” and “quarantine” (34). Another study created a codebook of online English-language anti-vaccination narratives and rhetoric and identified the nine most used codes. They were “Corrupt Elites,” “Vaccine Injury,” “Sinister Origins,” “Freedom Under Siege,” “Health Freedom,” “Think of the Children,” “Do Your Own Research,” “Heroes and Freedom Fighters,” and “Panic Button” from YouTube, Twitter, Facebook, and Instagram platforms (35). In addition, in Spain, the COVID-19 typology was identified by analyzing the science and health-related hoaxes that spread during the pandemic. This can serve as a preliminary framework for future research and can help develop systems for automated detection of health and science-related hoaxes. According to their connection to scientific knowledge, the four types were “hasty” science, decontextualized science, badly interpreted science, and falsehood without a scientific basis (36). Analysis of Facebook and Twitter posts in Finland helped develop a risk perception framework that included knowledge, perceptions, personal experiences, trust, attitudes, and cultural values that could be used as search terms to monitor public risk perception in future pandemics and to inform formulating effective messages (37).

In Russia, analysis of text from social media was used to model the detection of social stress in users. It used a neural network and linguistic analysis methods to assess users’ perception of government actions and identified points of tension in matters of communication during emergencies. It aims at improving the interaction between the government and society and to timely adjust government plans and actions to ensure resilience in emergencies for public health purposes (38).

Online surveys and analysis of epidemiological data were implemented in high-income countries such as Hong Kong, Japan, South Korea, Singapore, the UK, the USA, Italy, and New Zealand. These surveys aimed to investigate the relationship between infodemic with vaccine willingness and uptake, the strictness of public health and social measures, COVID-19 vaccine coverage, and health literacy (39–41).

Digital tools and technologies were used to address the challenge of synthesizing unsettled science and informing science translation and communication. EpidemiXs has been used by 30 health institutions in Spain, and a novel ecosystem of digital tools centralizing official and validated information on COVID-19 for health workers and the public in a single hub. EpidemiXs reached 1 million users and 2 million views in March 2020. It served as an evidence aggregation and science translation function, covering over 150 COVID-19-related studies in easy-to-understand and user-friendly formats. This made the scientific evidence more accessible to the public (42). In another example, Illinois-based medical professionals developed the IMPACT amplifier to facilitate interdisciplinary discussion and coordinate action. This tool allows the dissemination of accurate medical information and debunks misinformation while minimizing harm related to personal and professional harassment that can come with social media advocacy (43). In addition, the UK National Institute of Health and Clinical Excellence (NICE) in the UK adopted three automation approaches to evidence review and synthesis to facilitate faster processing of the new COVID-19 evidence in the production of surveillance guidelines. This approach demonstrated that human analysts accepted the assistance of machine-learning technology and showed that the approach was as good as using human analysts in the evidence search and synthesis process (44).

This study has several limitations. As health authorities and other sectors of society responded to the global COVID-19 pandemic and effects of the infodemic in their communities, much of the experience and knowledge that was gained from the response still needs to be evaluated and reported. Close to 4 years after the start of the COVID-19 pandemic, there are still gaps in the evaluation and reporting of the experience from national health authorities and other organizations working in health. This gap is apparent when comparing reporting of infodemic management projects at WHO infodemic management conferences, at conferences of national and regional public health association’s or on social and behavior change, digital society, health communications, or broader complexity science, misinformation, or epidemiological topics, for example. Challenges in capturing this arose due to the dynamic nature of the COVID-19 epidemiology globally and locally. As seen in the transition from public health and social measures to manage the pandemic, to the introduction of vaccines. Another example is, the refocus of the health systems to restoration of essential health services and programs while dealing with the impact of the pandemic on the essential health services, notably the burnout of health workers. Furthermore, the changing information environment in relation to attempts to regulate digital platforms and counter hate speech along with technologies like generative AI, contributed. As did the effects of pandemic fatigue on the attitudes of populations in relation to recommended health guidance. These continue to be challenges most health authorities struggle with today. As the information environment, epidemiology, health system priorities and capacities were changing, so did the actions and strategies used. This might have additionally slowed the evaluation and reporting of strategies and interventions used for infodemic management. This systematic review captures a snapshot of the evidence as available at this time and shows the need to systematically capture the evolution of evidence reported and generated. Such rapidly growing fields of research and practice are an example for establishment of living literature reviews that are updated regularly. This has also been recognized by the WHO as a process of setting up a structure for a living evidence gap map on infodemic management interventions (27).

Because the field is so new, it is also possible that this review might have missed studies that were not using the keywords that the field is using today, but rather were published in with the language and frameworks that are specific to their scientific discipline. For example, health promotion and commercial determinant of health, digital sociology, participatory action research, health literacy, information science and information related behaviors. Also those in topics tangential to health and infodemics, such as climate change misinformation, and misinformation during elections, cybersecurity, or health equity. Consequently, the studies that were included in this review do not cover the complex online-offline information environments (45, 46), and focus on social media and text messaging instead of social relationships, designed environments, and differentials of impact of content in different communities (47–49), and miss the person-centric understanding of what kind of information did they have (13, 50).

Infodemic management is a public health practice that has supported the response to the COVID-19 pandemic and other outbreaks since 2020, such as Ebola, diphtheria, mpox, measles, and polio. It is likely many interventions and practices that have been used in the field have not yet been reported in the literature by practitioners; this is evident by the number of reports from the field and from many countries and health authorities globally that presented and participated in WHO infodemic management conferences, but that has not yet been reported in the research literature. Moreover, the evaluation frameworks related to health information and health behaviors in the scope of infodemic management are still in development and are difficult to implement, which may have also contributed to the lag in publication. The WHO infodemiology research agenda emphasized implementation research and human-centered design approaches to speed up the generation of knowledge based on infodemic management interventions and strategies, as well as their transferability across health topics and contexts.

Moreover, the included studies showed the diversity of focus in the components of the infodemic (some focusing only on misinformation, or disinformation, on the changing scientific knowledge base, on people’s questions, etc.), or on either online or offline spaces. Because the infodemic phenomenon is so complex and encompasses the entirety of the information environment’s interaction with the health system, future work might consider reporting the focus of the study as an attribute in the analysis.

## 5 Conclusion

Most of the infodemic management interventions in this study implement a simple understanding of the WHO infodemic management framework which has itself rapidly matured over time since 2020. Future investments, strategies, and interventions should empower health authorities and health workers to apply the evidence-based and risk assessment to monitoring, detecting, and intervening on infodemic challenges, as well as learning from the experience and strengthening the systems to improve operations and develop more mature infodemic management systems and strategies. Moreover, a strong infodemic management function in a health authority at national and subnational level will promote better recognition of infodemic and misinformation. It will inform the delivery of communications, engagement, services, and interventions that are acceptable and usable by communities they serve. Some resources from the WHO that can help build capacity in the workforce and plan integration of infodemic management into routine processes are the WHO/UNICEF manual on how to build an infodemic insights report (51), an OpenWHO infodemic management eLearning channel (52), and the WHO competency framework for building a workforce to manage infodemics (12).

Strengthening health and digital literacy, engaging and empowering communities via participatory design, implementation and evaluation methods therefore are a priority. The COVID-19 infodemic was a great leveler; no one country mitigated the harmful effects of the COVID-19 infodemic easily. International collaboration, new partnerships across parts of society, and risk-based interventions and policies by health authorities are needed to tackle this. As declared in the hosted Riyadh Global Digital Health Summit, developing a resilient infodemic management plan, and creating curricula to elevate workforce skills and capabilities is urgently required.

## Author contributions

LA: Conceptualization, Writing – review & editing, Supervision, Data curation, Formal analysis, Investigation, Methodology, Software, Validation, Writing – original draft. TP: Conceptualization, Writing – review & editing. CT: Writing – review & editing, Data curation. ZA: Writing – review & editing, Data curation. ED: Writing – review & editing, Conceptualization. SR: Conceptualization, Writing – review & editing, Supervision.
