# Supplementary material for: Management of infodemics in outbreaks or health crises: a systematic review
Source: Front Public Health. 2024 Mar 15;12:1343902. doi: 10.3389/fpubh.2024.1343902 (PMC10986759; doi:10.3389/fpubh.2024.1343902)
Supplement: Supplementary file 1 [file Data_Sheet_1.PDF]

Supplementary Material

Supplementary Table 1 Extraction table for included studies.

Referen-ce	Type of Study	Type of outbr-eak	Source of information caused infodemics (Digital/Physical Environme-nt)	Impact: e.g. Change in health behaviour and/or Epidemic management	Duration (inc, date of intervent-ion)	Aim	Targeted population / Country	Infordenics management strategy applied	Outcome	Effectiven-ess Assessed Yes/No	Recommend-ation	Quality Appraisal
Abbas et al, 2021	Review	COVID-19	Newsfeeds and posts on social media	Increased global mental health risk		To explore how educating people through social media platforms can help reduce the mental health consequences of COVID-19 to manage the global health crisis	Global and Pakistani people	Using the non-pharmaceutical interventions (NPIs) approach:<br><br>launched special transmissions in different media channels and invited health professionals to provide useful information on the pandemic COVID-19	Reduced the critical situation of the COVID-19 outbreak for the people's well-being	yes		Moderate

Akpan et al, 2021	infodemiology study	SARS-CoV-2 and COVID-19	Web		first 6 months after the SARS-CoV-2 outbreak (1 January – 30 June 2020)	To evaluate the use of web searching to learn about SARS-CoV-2 and COVID-19	Global	Used Google Trends' worldwide search index	The keywords that people use to search the web to learn about the ongoing COVID-19 pandemic arev'pastCoVepidemics' and presCoVpandemic keywords.<br><br>The predictor of people's behaviour toward public health measures were "social distancing," "wash hands," "isolation," and "quarantine".	Yes		High

Al-Aghbari et al 2023	Cross-Sectional study	COVID-19	Social media	Physical harm: limited, accurate knowledge about available treatments, misplaced actions, and dismissal of proven public health measures<br><br>Social harm: victimization, stigma, and violent aggression among community members<br><br>Political harm: limited trust in officials, rejection of official guidelines, disregarding of government-led responses.		To assess the compliance with preventive measures and investigate the role of infodemics in people's non-compliance with COVID-19 containment measures in Yemen	Yemen	A mixed method approach in which raw aggregated data were taken and analysed from multiple sources (COVID-19 Government Response Tracker and Google COVID-19 Community Mobility Reports), and in-depth interviews.	The population in Yemen complied with the governmental containment measures at the beginning of the pandemic<br><br>Low government transparency, spread of misinformation and lack of access to reliable sources reflects negatively people believe in COVID-19 and compliance in<br><br>Contains preventive measures	Yes		High

				Psychological harm: mental health disorders due to extreme anxiety and panic					and creates social pressure on those who showed compliance with the WHO guidelines.			
Albrecht et al 2022	experimental study: social media – based science communication project	COVID-19	Social media	A spread of misinformation quickly	2 years since March 2020	To disseminate trustworthy, comprehensive, and timely scientific content about the pandemic to lay audiences via social media<br><br>To promote media literacy and information-	Global	Dear Pandemic is an innovative, multidisciplinary, social media–based science communication project. The volunteer team of scientists and information per week on pandemic-relevant topics for 2 years.	*Dear Pandemic Project:*<br><br>*1- Foster trust*<br><br>*2- Fight the Infodemic on Its Own Turf*<br><br>*3- Be Specific in providing advice on reducing stress, anxiety and depression*<br>*4- Make It Painless: it makes posts easy to read and includes 1*	Yes		Moderate

						hygiene practices, equipping readers to better manage the COVID-19 infodemics within their own networks.			*key takeaway at the top.*				
Alknawy et al 2023	protocol	COVID-19				A call to action to create the infrastructure needed to share effective digital health evidence-based practices and high-quality, real-time data locally and globally to provide actionable information to more health	Kingdom of Saudi Arabia<br><br>Global	The Riyadh Declaration on Digital Health was a call to action	A proposal for robust digital public health leadership and nine key recommendations for data and digital health to address and develop preparedness infrastructure for future pandemics and health threats	Yes	Working towards reaching a consensus on which areas to prioritise to achieve digital transformation in an equitable, inclusive and sustainable manner	N/A	

						systems and countries			The key requirements for a robust Digital health infrastructure are:<br><br>- developing contextually sensitive communication strategies<br><br>- instigating infoveillance (information monitoring) capabilities with fact-checking underpinned by legal standards technology and data governance to support			

									standardisation<br><br>- a global minimum dataset to guarantee data usability and integrity based on agreed standards; and education providers and industry working closely to co-create meaningful curricula after establishing gaps in staff skills and capabilities.			
Aslani et al, 2022	Review Article	COVID-19		Social hazards, improper health behaviour,	The database searched up to April 2022	To determine the infodemics challenges of	Global		Strategies to deal with COVID-19 and other similar	Yes		Moderate

				and scientific hazards		COVID-19 and the strategy to deal with them			conditions are:<br><br>- active confrontation with centres and scientific sources<br><br>- effective intervention of health care professionals, responsible participatory actions<br><br>- actions of governments and authorities<br><br>- monitoring and identifying incorrect information<br><br>- heightening people's awareness			

									- encouragement for vaccination<br><br>The level of health literacy of individuals in society in developed and developing countries at the beginning of the pandemic was low.			
Bas-Sarmiento et al, 2022	Cross-sectional study	COVID-19	News, radio and TV talk shows, press conferences, national press, social media, the internet, the Ministry of Health, the Consumer Affairs and Social Welfare website			To evaluate the level of Health literacy related to COVID-19, risk perception, misinformation, and the attitudes and behaviours adopted	499 Spanish students	Evaluation of the level of Health literacy related to COVID-19 by using online “Google Forms” questionnaire distributed via WhatsApp and social networks	- 78.4% (n = 391) of university students had difficulty distinguishing whether a news item or information about COVID-19 was true or false	Yes		

			(MHCSW), and the WHO			to prevent the spread of coronavirus in Spanish university students.			- 63.8% had an inadequate level of Literacy related to COVID-19.<br><br>They practised a mean of 7.54 out of 9 preventive behaviours<br><br>- The level of Health Literacy is correlated with the adoption of preventive measures			
Bertinato et al, 2021	Case study	COVID-19	pre-print and peer-reviewed papers		March-May 2020	To provide a rapid technical and scientific advice to the State	Health workers in Italy	An ad hoc Working Group on Scientific Literature updates (WG SL) was set up at ISS to	- The WG SL screened 4,568 pre-prints and 15,590 peer-	Yes		High

						and Regions during Sars-CoV-2 pandemic prepared ness by the Istituto Superiore di Sanità (ISS)		screen pre-prints and peer-reviewed papers from arXiv, medRxiv, bioRxiv, and Pubmed to provide real-time knowledge and empirical evidence addressed to health workers.	reviewed papers<br><br>- Promoting health literacy, with a cross-cutting approach is a powerful heritage of Public Health Institutes and a proven effective non-pharmacol ogical interventio n:<br><br>The interdiscipli nary activitypro ved that quick sharing of the scientific knowledge with health workers			

									during the pandemic can be eligible among readiness tasks for the future pandemic challenges				
Chen et al, 2022	Cross-sectional study	COVID-19			Jun-21	To examine the effect of perceived information overload (IO) and misinformation on vaccine willingness and uptake within a cross-national context<br><br>To investigated how trust in multiple	residents and representatives of the general population aged ≥18 in six Asian and Western jurisdictions including Hong Kong, Japan, South Korea, Singapore, the UK, and the US.	A cross-national online survey	- Trust in the government and civil societies tended to strengthen the positive effect of IO and reduce the negative impact of misinformation on vaccine willingness and uptake<br><br>- The relationship between belief in misinforma	Yes		Moderate	

						institutions affected vaccine outcomes and moderated the relationship between the infodemics and vaccine attitude and behaviour			tion and getting vaccinated against COVID-19 was unexpectedly stronger among those with a higher level of trust in healthcare professionals.			
Etta et al, 2022	observational: Comparative descriptive study	COVID-19	Social Media	Leading to harmful decisions for society and a severe threat to information security, public health and democracy		To assess the interplay between the infodemics and specific aspects of the pandemic, such as the number of cases, the strictness of containment measures, and the	Italy, the United Kingdom, and New Zealand	Performing a comparative study on three countries that employed different management of the COVID-19 pandemic in 2020—namely Italy, the United Kingdom, and New Zealand, by conducting:<br><br>- Analyses of the three countries from an epidemiologic	- Posts referring to reliable sources are consistently predominant in the news circulation,<br><br>- Users engage more with reliable posts rather than with posts referring to	Yes	factors related to the epidemiological and informational ecosystems can serve as proxies to assess the evolution of the infodemics	High

						news media coverage.		al perspective to characterize the impact of the pandemic and the strictness of the restrictions adopted.<br><br>- collecting a total of 6 million posts from Facebook to describe user news consumption behaviours with respect to the reliability of such posts<br><br>- quantifying the relationship between the number of posts published in each of the three countries and the number of	questionable sources			

								confirmed cases, the strictness of the restrictions adopted, and the online news				
Feinberg et al, 2022	Cross-sectional survey	COVID-19	Massive amounts of COVID-19 information, misinformation, and disinformation flooding the airwaves, print sources, and the internet	Decrease uptake of vaccine among people with lower health literacy		To understand health literacy levels within Fulton County, Georgia, US and their relationship to vaccine prevalence	US: Fulton county residents ages 18 and over (n = 425)	Completing an online Health Literacy Questionnaire	- There were statistically significant variations in vaccine prevalence $\chi2(3) = 29.325$, $p < 0.001$ among the three county areas<br><br>- The lowest-resourced county area had the lowest vaccination prevalence and health literacy rates	yes		Moderate

Gisondi 2022	Study intervention	Covid-19	Social Media	Increasing vaccine hesitancy among the public, particularly in vulnerable communities, which persists today	Aug-21	To develop best practices for social media companies to mitigate online misinformation and disinformation.	- The conference speakers were the study participants, and transcripts of their presentations were the data for this study.<br><br>- A total of 26 participants spoke at the virtual conference and represented a wide array of occupations, expertise, and countries of origin.	INFODEMIC conference: A Stanford Conference on Social Media and COVID-19 Misinformation (INFODEMIC)	There were common themes in the participant remarks:<br><br>- improving trust in science and medicine, promoting equity in vaccine access and healthcare<br><br>- identifying social media best practices, and creating inter-organizational partnerships.	Yes	The conference offered specific recommendations that social media companies, healthcare professionals, and the general public can adopt to better mitigate online health misinformation.	Moderate

Hughes 2021	Experimental study	COVID-19	Social Media	Vaccine hesitancy		To use qualitative coding methodology to identify salient narratives and rhetorical styles common to anti-vaccine and COVID-denialist media		To create a codebook of online English-language anti-vaccination narratives and rhetoric, so as to support government officials and civil society groups engaged in managing disinformation during the COVID-19 vaccination campaign	- Most frequent were narratives centred on “corrupt elites” and rhetorics appealing to the vulnerability of children<br>- Of the final codebook’s list of narrative tropes, five patterns represented more than half of the coded items: “Corrupt Elites,” “Vaccine Injury,” “Sinister Origins,” “Freedom Under Siege,” and “Health Freedom.” Of the final codebook’s	Yes	The identification of narratives and rhetorics codes may assist in developing effective public health messaging campaigns since narrative and emotion have demonstrated persuasive effectiveness in other public health communication settings	High

									list of rhetorical styles,<br><br>- four patterns represented just over 40% of all coded rhetorical items: “Think of the Children!” “Do Your Own Research,” “Heroes and Freedom Fighters,” and “Panic Button”<br><br>All nine of these codes appeared across all four major platforms from which data were sampled:			

									YouTube, Twitter, Facebook, and Instagram.			
Lemaire 2022	Evaluation study	COVID-19	Web, TV, Studies		March 2020	To foster collaboration between researchers, institutions and individuals to promote “open data” in order to enrich the scientific community and further accelerate science in the fight against COVID-19	30 healthcare institutions in Spain	EpidemiXs is an innovative ecosystem of digital tools centralizing official and validated information on COVID-19 for healthcare workers and the general public in a single hub	EpidemiXs was reached 1 million users and 2 million views<br><br>- EpidemiXs Studies dissemination platform has published and shared the work of over 150 COVID-19 related studies in easy-to-understand and user-friendly formats, making the studies and their work more accessible by the	Yes		High

									general public and driving engagement			
Leon et al, 2022	Qualitative study	COVID-19				To analyze the science and health-related hoaxes that were spread during the pandemic with the objectives of (1) identifying the characteristics of the form and content of such false information, and the platforms used to spread them, and	Spain	Analysing of the science and health-related hoaxes of COVID-19 that spread during the pandemic	- Science and health content played a prominent role in shaping the spread of the hoaxes during the pandemic<br><br>- The most common hoaxes on science and health involved information on scientific research or health management, used text, were based on deception, used real sources,	Yes	This typology can serve as a preliminary framework for future research and can help develop systems for automated detection of health and science-related hoaxes.	High

						(2) formulating a typology that can be used to classify the different types of hoaxes according to their connection with scientific information			were international in scope, and were spread through social networks<br><br>- The study identified four types according to their connection to scientific knowledge: "hasty" science, decontextualized science, badly interpreted science, and falsehood without a scientific basis			
Lohiniva et al, 2022	Qualitative study	COVID-19	Facebook and Twitter	risk communications	March-May 2021	To identify and describe factors related to COVID-19 risk	Finland	The study identified concepts linked with the pandemic risk perception that included knowledge,	- lack of knowledge increases pandemic-related risk perceptions		The risk perception framework can be used as a search terms to monitor public risk	

						perceptions of the public in Finland<br><br>To make this information readily available to those who communicate with the public during crises		perceptions, personal experiences, trust, attitudes, and cultural values.<br><br>The study was based on a dataset of over 10,000 comments on the Facebook and Twitter posts of the Finnish Institute of Health and Welfare (THL)	- the pandemic risk perception is linked with various perceptions that can be addressed when aiming to reduce the fear surrounding COVID-19<br><br>- mistrust can easily generate a great deal of fear and anger<br><br>- negative personal experiences increased risk perception whereas positive		perception in future pandemics and epidemics.<br><br>The framework will be particularly beneficial for risk communicators and other public health officials who can utilize the framework to formulate effective messages and other risk communication content at the right time during future pandemics and epidemics.	

									experiences decreased it<br><br>- individual risk perception is not only linked with individual factors but also with broader sociocultural values, such as vertical culture and individual rights<br><br>- vertical culture increases the risk perception towards environmental threats			
Moretti et al 2023	Pre-post study	COVID-19	Digital	Spread of uncontrolled health information	A pre-post study was conducted at the University of	To investigate the Digital Health Literacy	Italy- First Medical year students at the University	This course focuses on assessing the quality of medical information	- Almost all students (94.5%) valued the "dottorem aeveroche"	Yes	Effective tools and resources such as the DMEVC website	Moderate

				and fake news	Florence between November and December 2020	(DHL) skills of Italian medical students and the effectiveness of an informatics course offered by the University of Florence (Italy)	of Florence participated in a web-based survey before and after attending the informatics course	using the "dottoremaeveroche" (DMEVC) web resource offered by the Italian National Federation of Orders of Surgeons and Dentists, and on health information management.	DMEVC as an educational tool<br><br>-The DMEVC tool was effective in improving medical students' DHL skills		should be used in public health communication to facilitate access to validated evidence and understanding of health recommendations	
Munoz-Sastre et al 2021	Case study	COVID-19	Media and social networks	Dissemination of disinformation	From 9 November 2020 (when the 73rd World Health Assembly resumed) to 14 March 2021 (three months after the start of vaccination)	To explore how the WHO uses its Twitter profile to inform the population about vaccines against the coronavirus	WHO Tweets<br><br>849 vaccine-related tweets posted by the WHO on its Twitter account	The results were compared with the actions carried out by the WHO and with the information and debates throughout this period		Yes	The WHO is decidedly committed to the use of these tools as a means to disseminate messages that provide the population with accurate and scientific information, as well as to combat mis- and disinformation about the	Low

											SARS-CoV-2 vaccination process	
Nyoni et al 2022	Review of litrature	Covid-19				To justify effective knowledge management as a precursor for mitigating the effects of a crisis, Covid-19 pandemic in particular, through key antecedents of leadership, culture, and information and communication technology (ICT)			- sustainable knowledge management during the Covid-19 crisis largely depends on a decisive leadership style that puts employees at the centre<br><br>- a culture that embraces knowledge as a core asset, and supportive ICT infrastructure.<br><br>- developme	Yes		Low

									nt of a proposed model understanding further the relationships between the key determinants of leadership, culture, ICT, and knowledge management during the Covid-19 crisis			
Park et al 2021	experimental study	Covid-19	News articles and social media		from 1st January to 15th May 2020)	To examines the social orchestration of COVID-19 in South Korea (hereafter, K-COVID-19) during the first wave (from January 1st to	South Korea	Utilities of Integrated Information Surveillance System (IISS) using empirical data from the first wave of COVID-19.<br><br>IISS is an à-la-carte infodemics surveillance solution that enables users	Measuring discourse congruence allows to gauge the distance between the discourse corpus from different sources, which can highlight consensus and	Yes	adequate and appropriate pandemic risk management, particularly at a global scale, should involve a larger and inclusive expert community and secure diverse	High

						May 15th, 2020) of the virus		to gauge the epidemic-related consensus, which compiles epidemic-related data from multiple sources and is equipped with various methodological toolkits – topic modelling, Word2Vec, and social network analysis.	conflicts in epidemic discourse. Furthermore, IISS detects discrepancies between social concerns and main actors.		communication channels	
Pattison et al 2022	experimental study	Covid-19	Google and Web-based text ads	Overabundance of information on COVID-19	Between 11 September 2020 and 23 November 2020	To understand the way in which ad information is worded for the public leads searchers to click through to WHO content.	WHO tested 71 text ads in English across four COVID-19 topics using a mix of message frames: descriptive, collective, gain, loss, appeals to values and emphasising reasons	To disseminate accurate and timely information to counteract misinformation and disinformation that has arisen as part of an 'infodemic'	- There were 13 million views of the experimental WHO text ads leading to 1.4 million click-throughs to the WHO website<br><br>- Optimising	Yes	Similar collaboration between big technology companies (e.g google) and governments and global health agencies has the potential to advance public health.	High

									message framing in English language text ads informed by behavioural science was shown to be an effective mechanism for connecting millions of users to WHO content for messages on COVID-19<br><br>- The best-performing framings were more than twice as effective as the worst-performing framings (18.7% vs 8.5%			

									engageme nt rate)<br><br>- National and global health practitione rs may be able to apply the message tactics<br><br>- WHO found to be successful in rapidly testing and optimising messages for more successful public health campaigns			
Purnat et al 2021	experi mental study	Covid-19	Public online conversatio ns		23 March 2020	To use a public health infodemic intelligen ce analysis methodol	This Public online conversati on research framework is used in	Development of a public health social listening taxonomy, a structure that can simplify the chaotic	This methodolo gy produced infodemics digital intelligence , providing	Yes	The expansion of the methodology across regions will allow to compare the	High

						ogy for weekly analysis of digital media data to identify, categoriz e, and understan d the key concerns expressed in online conversat ions	Canada and is piloted at the national level in South East Asia, Western Pacific and Africa.	information ecosystem to enable an adaptable monitoring infrastructure that detects signals of fertile ground for misinformatio n and guides trusted sources of verified information to fill in information voids in a timely manner.	WHO with week-on-week informatio n to prioritize actions to fill informatio n voids with high-quality health informatio n and inspire new ways of thinking and communica ting risk during health emergenci es		similarities and differences of how this insight can be used at the local level and add the layer of multilingual content analysis.	
Purnat et al 2021	Case study	Covid-19	social media	Challenging effective response	January 2021 c	The aim of the platform is to better integrate social listening	Data is collected daily from online COVID-19 conversati ons in English,	The WHO Early AI-supported Response with Social Listening (EARS) is a platform that	The dashboards show how the topics of conversati on change and evolve	Yes	Three research projects will be carried out to deep dive into development	High

						with other data sources and analyses that can inform infodemic response.	Spanish, French and Portuguese from publicly available sources (ex Twitter, online forums, news comments, and blogs).	summarizes real-time information about how people are talking about COVID-19 in public spaces online in 20 pilot countries and in four languages.	country by country over time, such as: what are the most popular categories and those gaining traction, and their patterns; what are the top and rising terms and hashtags within each category; what are the differences in conversations by; the composition of the conversation by intention: questions (confusion), complaints (frustration) or praise.//The EARS platform		of new measurements and measure dimensions.	

									provides a public social listening tool from public online sources, that can be combined with other COVID-19 datasets for better integrated analysis to support infodemic response in countries.			
Raskhodchikov et al 2023	Cross-sectional study	Covid-19	Social Media		third wave of the COVID-19 pandemic in Russia	To analyse the information media environment and communications behaviour of actors during the third wave of the COVID-19	Population in Russia	Analysis of Opinions in Social Media<br><br>The application of an interdisciplinary approach using network analysis of texts and sociological research. A model for	- The validity of the results of the analysis of social network data was verified using a sociological survey.<br><br>- This approach	Yes	The method of detecting social stress in text messages in social networks provides opportunities for qualitative analysis of social network data.	High

						pandemic in Russia<br><br>To assess citizens' perceptio n of governme nt actions.		detecting social stress in the textual communicatio n of social network users using a specially trained neural network and linguistic analysis methods	allows us to identify points of tension in matters of public health promotion, during crisis to improve interaction between the governmen t and society, and to timely adjust governmen t plans and actions to ensure resilience in emergency situations for public health purposes.		Fixing the growth of stress in network communicati on can be used not only in crisis but also in normal times to identify social conflicts and spread false information on public health issues.	
Royan et al 2022	review	Covid-19				To discuss IMPACT amplifier work within the following broad themes: rapid	This group of Illinois-based medical profession als, science communic ation experts,	IMPACT (Illinois Medical Professional Action Collaborative Team) is a 501(c)(3) non-profit	it is imperative for medical profession als to utilize all available tools to disseminat	Yes	Understandi ng the successful use of health professional amplifiers is a substantial way that	Moderat e

						dissemination and promotion of accurate medical information and public health guidance, combating disinformation, and countering harassment.	and researchers within IMPACT have used the IMPACT amplifier to facilitate interdisciplinary discussion and coordinate action	organization designed to help physicians and health professionals engage in grassroots networks, advocate for evidence-based solutions, advise influential stakeholders, and amplify solutions to protect individuals and communities across the state.<br><br>IMPACT's Twitter account, @IMPACT4HC, is a verified account with 3232 followers<br><br>The goals of an amplifier	e accurate medical information and combat disinformation while minimizing harm related to personal and professional harassment that can come with social media advocacy.		physicians and other public health professionals can achieve this essential goal<br><br>The future directions include expanding beyond COVID-19 to other public health topics, such as gun violence and reproductive justice	

								are to connect people, collaborate, strategize, and amplify ideas on Twitter and other social media platforms.				
Sood et al, 2022	AI resear ch article	Covid1 9	Digital		Mar 2022	To facilitate faster processin g of increased COVID-19 evidence	England & Wales	Developed three automation strategies employed by the National Institute for Health Care Excellence (NICE) to facilitate the Covid19 evidence processing	Based on testing the three automatio n approaches adopted by NICE to develop guideline surveillanc e were effective in managing increased numbers of Covid19 evidences. It overcame the hesitancy of using machine learning and the idea that the latter approach might miss	yes	no clear recommenda tions	Moderat e

									important studies			
vanderLinden 2022	Review	COVID-19				To summarize what we know along three key dimensions of the infodemic: susceptibility, spread, and immunization		Lists possible interventions including misinformation debunking	Debunking is the best strategy to protect public health from misinformation<br><br>The spread of misinformation has undermined public-health efforts, from vaccination uptake to public compliance with health protective behaviours.	N/A	Further research is also encouraged to outline the benefits and potential challenges of applying the epidemiological model to understand the psychology behind the spread of misinformation.	Moderate

White et al, 2023	Research article	COVID-19	AI study		December 2020 till October 2022	To implement regular internal processes to understand the needs of those using the EARS system	N/A	The WHO Early AI-Supported Response with Social Listening (EARS) platform was developed to help inform infodemic response during the COVID-19 pandemic by providing useful information and actionable insights<br><br>EARS uses Artificial Intelligence (AI) and machine learning to categorize publicly available digital and social media data to a public health taxonomy	A new sophisticated 'social indictors' panel helps users to identify social change in narratives across categories such as distrust, civic unrest, or polarization.<br><br>An added reporting feature allows users to create, store and collaborate on producing reports, including options for automation.<br><br>An additional	yes		High

								Technical review was conducted each month with the development team to review global trends, the public health taxonomy, data sources and data volume.	five languages and 10 countries were added, and iterations made to the taxonomy.<br><br>To enable more informed narratives about the COVID-19 vaccine roll-out, a new dashboard interface was added.<br><br>The EARS helped public health to be informed of free, real-time access.			

									According to the end user feedback and evaluation, the EARS stayed useful, informative and relevant			
White et al, 2023	Research article	Covid19	Posts from social media		March 2020 & October 2022	To analyse the social media posts	Global	Classified the narrative trends into categories showed how people with time change their attitude towards the infection<br><br>Publicly available social and news media data is collected from Meltwater and CrowdTangle on a weekly basis in English,	It helped the endodermic manager to translate the classified information into a recommendation<br><br>Supported the infodemic manager about the trend of narrative<br><br>The three key periods associated with high	N/A	N/A	Low

								French and Spanish.<br><br>These data are categorized to a public health taxonomy which has 5 overarching categories (the cause of the virus, the illness, the treatment, the interventions and perceptions on information) and 42 sub-categories	data volumes were: A peak in March – April 2020. in October 2020 and between December 2021 and January 2022<br><br>- There were discussions about travel restrictions, severity of Omicron, and vaccination, as well as frustration at the spread of vaccine.			

Supplementary Table 2 Summary of implemented infodemic interventions from included studies.

Authors	Implemented Interventions
Abbas et al., 2021 (31)	Launch special transmissions on TV channels and invite health professionals to provide accurate updated information
Akpan et al., 2021; Hughes et al., 2021, León et al., 2022, Lohiniva et al., 2022, Al-Aghbari et al., 2023, Bertinato et al., 2021, Purnat et al., 2021a, Nyoni and Kaushal, 2022, Van Der Linden, 2022 (28, 29, 32-36, 52-55)	Use web and scientific databases such as Google Trends' worldwide search index and search keywords and predictors of people's behaviour toward public health measures identification
Albrecht et al., 2022, Muñoz-Sastre et al., 2021 (56, 57)	Post science-based COVID-19 information via the web, social media, and channels
Al-Aghbari et al., 2023 (32)	Conduct in-depth interviews to complement and verify the aggregated social media data analyses
Chen et al., 2022, Etta et al., 2022, Feinberg et al., 2022, Moretti et al., 2023, Pattison et al., 2022 (38-40, 58, 59)	Implement online surveys and analyse epidemiological data to examine the effect of infodemics on vaccine uptake willingness and strictness of containment measures
AlKnawy et al., 2023, Gisondi et al., 2022 (30, 60)	Announce calls to action and declarations on digital health and conduct conferences
Aslani et al., 2022, Bas-Sarmiento et al., 2022 (61, 62)	Explore challenges and strategies for infodemics
Royan et al., 2022, Lemaire et al., 2022, Raskhodchikov and Pilgun, 2023, Sood	Create an innovative ecosystem of digital tools to validate information on COVID-19, such as Epidemics, using a model for detecting social stress in the textual communication of social networks to assess citizens' perception of government actions, using IMPACT amplifier to facilitate

et al., 2022, White et al., 2022, Purnat et al., 2021c (37, 41-43, 63, 64)	interdisciplinary discussion and coordinate action or Early AI-Supported Response with Social Listening (EARS) which is a digital intelligence listening platform
Park et al., 2021 (65)	Utilise the Integrated Information Surveillance System (IISS) that uses empirical data from the first wave of COVID-19 to gauge the epidemic-related consensus, which compiles epidemic-related data from multiple sources and is equipped with various methodological toolkits such as topic modelling, Word2Vec, and social network analysis
